# Parametric resonance of Alfvén waves driven by ionization-recombination waves in the weakly ionized solar atmosphere

**DOI:** 10.1098/rsta.2023.0226

**Published:** 2024-06-09

**Authors:** I. Ballai, E. Forgács-Dajka, M. McMurdo

**Affiliations:** ^1^ Plasma Dynamics Group, School of Mathematics and Statistics,The University of Sheffield, Hicks Building, Hounsfield Road,Sheffield S3 7RH, UK; ^2^ Department of Astronomy, ELTE Eötvös Loránd University, Institute of Physics and Astronomy, Pázmány Péter sétány 1/A, Budapest 1117, Hungary; ^3^ HUN-REN–SZTE Stellar Astrophysics Research Group, Szegedi út, Kt. 766, Baja 6500, Hungary

**Keywords:** MHD, partially ionized plasmas, waves, instabilities

## Abstract

Parametric coupling of waves is one of the most efficient mechanisms of energy transfer that can lead to the growth or decay of waves. This transfer occurs at frequencies close to their natural frequencies. In partially ionized solar plasma, there are a multitude of waves that can undergo this process. Here, we study the parametric coupling of Alfvén waves propagating in a partially ionized solar plasma with ionization-recombination waves identified by our study to appear in a plasma in ionization non-equilibrium. Depending on the parameters that describe the plasma (density, temperature), coupling can lead to a parametric resonance. Our study determines the occurrence conditions of parametric resonance, by finding the boundaries between stable and unstable regions in the parameter space. Our results show that collisions and non-equilibrium recombination can both contribute to the onset of unstable behaviour of parametrically resonant Alfvén waves.

This article is part of the theme issue ‘Partially ionized plasma of the solar atmosphere: recent advances and future pathways’.

## 1. Introduction

The temperature of the lower part of the solar atmosphere (photosphere and chromosphere) is a few thousand K, meaning that the plasma (for simplicity, here assumed to be purely hydrogen plasma) is in a partially ionized state, where electrons, positively charged ions (protons) and neutral particles can interact with each other through collisions. Collisions are a very effective way to exchange momentum and energy and for the thermalization of the plasma. Research in the dynamics of partially ionized plasmas, over the past decade, has undergone a significant increase driven by high-resolution observations and numerical investigations (for a comprehensive review of the observations and modelling of dynamics in astrophysical partially ionized plasmas see, e.g. [1]).

The framework in which dynamics in a partially ionized plasma is described depends on the frequency regime of interest. In particular, for dynamical changes for which the spatial scales are of the order of the mean free path of particles and temporal scales are of the order of the collisional time between particles (therefore, velocities are of the order of thermal velocity), one has to employ a framework where the equations that describe the dynamical evolution of the plasma contains information about the interaction between the fluids that make up the plasma. Since the coupling of electrons and ions in the solar chromosphere is much stronger than the coupling with neutrals [2], it is often enough to treat the plasma as a two-fluid system (charged particles and neutrals) and the frequencies of interest would be of the order of the ion-neutral collisional frequency. The equations that describe the state of the plasma and the associated waves have been discussed earlier by several studies, see, e.g. [1–8].

In many engineering, physical, electrical, chemical and biological systems, the oscillatory behaviour of the dynamic system owing to periodic excitation is of great interest. Such systems can exhibit a diversity of dynamic regimes with periodic, aperiodic and even chaotic oscillations. In general, the differential equations describing such systems are non-autonomous and solutions are obtained using, e.g. numerical methods, perturbation and averaging theory, point mapping methods [9].

When dynamics is driven by periodic excitation, the responses of physical systems can be categorized as forced oscillations and parametric oscillations. Forced oscillations appear when the dynamical system is excited by a periodic input. If the frequency of an external excitation is close to the natural frequency of the system, then the system will experience resonance, i.e. oscillations with a large amplitude. Parametric oscillations are the result of having time-varying (periodic) parameters in the system. In this case, the system could experience parametric resonance, and again the amplitude of the oscillations in the output of the system will be large. Current studies attempt to determine the occurrence conditions for parametric resonance, by detecting the boundaries between stable and unstable regions in the parameter space. In general, the secular growth of amplitude owing to parametric coupling between modes is suppressed by applying various renormalization techniques [10–12]; however fundamental physical processes can be recovered when studying the parametric coupling of waves since this process is often associated with amplification or decay of wave amplitude, to heating of the plasma or acceleration of particles.

Parametric coupling of waves has received special attention in the literature as one of the most effective ways to transfer energy and momentum between waves. In the solar wind, parametric coupling is believed to play an important role in the development of turbulent Alfvén wave cascades that can lead to instabilities that depend parametrically on the pump wave amplitude and the plasma beta [13–15]. The same parametric coupling between waves (although referred to as swing interaction) was studied in a series of papers in [16–18] and it refers to the parametric interaction of waves that results in the energization of waves, where the energy of non-electromagnetic origin transforms into the energy of electromagnetic oscillations.

The appearance of Kelvin–Helmzholz (KH) parametric instability in the presence of oscillatory flows at a tangential discontinuity has been proposed by [19] as a mechanism to extract energy from magnetohydrodynamic (MHD) kink waves in flux tubes, and to drive dissipation of this wave energy through turbulence. The variation of the Lagrangian displacement of the interface was given as a Mathieu-type equation, and two kinds of instabilities were recovered: a traditional KH instability and a parametric instability involving resonance between the oscillatory shear flow and two surface Alfvén waves. The latter occurs when the system is KH stable, thus favouring modes that vary along the flux tube, and as a consequence provides an important and additional mechanism to extract energy. The characteristic time scale for these instabilities was found to be around 100 s, for wavelengths of 200 km. The authors also found that the parametric instability is more likely to occur for smaller density contrasts and larger velocity shears, making its development more likely on coronal loops than on prominence threads. [20] used Si iv lines observed by the Interface Region Imaging Spectrometer (IRIS) in the transition region of a polar coronal hole to evidence parametric decay instability in the lower solar atmosphere. The power spectrum of density fluctuations near the solar transition region observed by these authors resembles the power spectrum of the velocity fluctuations but with the frequency axis scaled up by a factor of approximately 2. Their analysis also showed that the density fluctuations have a radial velocity of approximately $75\;\mathrm{km}\;s^{-1}$ and that the velocity fluctuations are much faster with an estimated speed of $250\;\mathrm{km}\;s^{-1}$. Their analysis suggests an interaction between sound waves and Alfvén waves in the transition region, which is evidence for the parametric decay instability.

In general, studies on the effect of partial ionization on wave propagation and the development of instabilities assume that the plasma is in ionization equilibrium, i.e. during the investigated temporal and spatial changes, the chemical composition of the plasma does not change, i.e. the processes of additional ionization and recombination are neglected. This assumption is very often violated, as the characteristic times for ionization and recombination are shorter or comparable with the temporal scales involved in the problem, e.g. the period of waves. Changes in the chemical composition of the plasma (in addition to temperature and pressure) are a way to bring the system to non-equilibrium [21]. The present study aims to investigate the nature and properties of non-propagating waves in a partially ionized plasma in ionization non-equilibrium, and the effect of the parametric coupling of these waves to Alfvén waves. The present contribution is structured as follows: in §2, we present the physical considerations that stay at the core of the studied problem as well as the mathematical background necessary to analyse the ionization-recombination waves. Section 3 is devoted to the investigation of the parametric coupling of ionization-recombination waves with Alfvén waves propagating in a partially ionized plasma, and we establish the conditions under which the coupling may lead to instability. Finally, our results are concluded and summarized in §4.

## 2. Physical consideration and mathematical background

In general, the standard way to study waves assumes that the equilibrium is stationary, or at least it varies over much longer time scales than any dynamical time scale of the physical problem. In a partially ionized plasma, the stationary equilibrium state is not always reached, as ionization of neutral atoms and recombination of ions and electrons often take place over time scales that are comparable with other temporal scales. That is why non-stationarity may be generated by atomic processes taking place in a plasma

Let us consider a partially ionized solar plasma made up of hydrogen atoms, protons and electrons. The physical extent of the plasma is much larger than the Debye radius, meaning that we have a quasi-neutral plasma in which the number densities of electrons and protons are equal. The collisions between these particles provide an ideal channel for momentum and energy transfer between species. According to standard solar atmospheric models (e.g. [22]) the ionization degree of the plasma (proportional to the ratio of the number density of neutrals to ions) varies between very large limits, from approximately $10^{4}$ in the solar photosphere to values of the order of unity at the top of the chromosphere. For our purposes, we assume that the plasma is weakly ionized, so our investigation is relevant to conditions we can find in the solar photosphere and lower chromosphere. In the solar corona, the plasma can be considered as being fully ionized.

It is well known that ionization and recombination are endotherm and exotherm processes, respectively. Equally, the ionization probability increases with the temperature of electrons, while lower-temperature electrons favour recombinations.

Since the degree of ionization is sufficiently small, the collisions of charged particles with neutral particles dominate over collisions between charged particles. In this limit, it is normal to assume that through ionization-recombination processes the variation of neutral number density is small compared to the total neutral number density. The ion temperature is assumed to be everywhere equal to the neutral gas temperature, and is therefore a constant. The electron temperature is assumed to be large compared with the neutral gas temperature; consequently, the neutrals are ionized only by collisions with the hot electrons and the dominant ionization and recombination processes are given by electron impact ionization and radiative recombination.

Initially, the high-temperature electrons will increase the ionization of the plasma, as the ionization probability increases with temperature. This process will lead to the cooling of electrons, which favour the recombination as the recombination rate increases with the decrease in temperature. Each recombination results in photons emitted that are absorbed by electrons via various processes (e.g. inverse Bremsstrahlung). According to the Langdon effect [23], the increase of electrons temperature applies mainly to cool particles increasing their temperature. As a result of this process, the distribution function of electrons tends towards a super-Gaussian distribution, which can transform into a Maxwelian distribution through mutual collisions of electrons. The enhanced temperature will lead, again to an enhanced ionization and the two processes repeat themselves. This cyclic variation of ionization and recombination leads to a non-propagating ionization-recombination wave resulting in a temporal change of the electron number density.

Assuming that the plasma is made up of single-level (bound) hydrogen, the temporal variation of the electron and neutral number densities as a result of ionization and recombination processes are given by

2.1 $$\frac{dn_{a}}{dt}=K_{R}n_{e}^{2}-K_{I}n_{a}n_{e}$$

and

**Figure 1.**
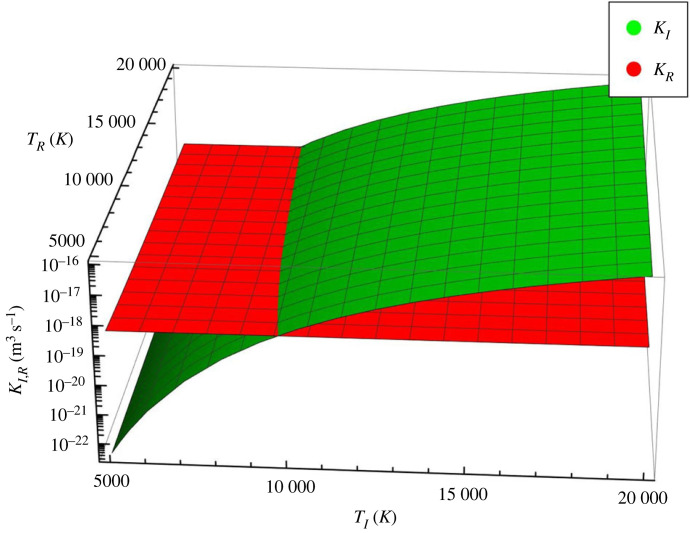
The variation of the ionization, $K_{I}$ (green surface), and recombination, $K_{R}$ (red surface), rates with temperature.

2.2 $$\frac{dn_{e}}{dt}=-K_{R}n_{e}^{2}+K_{I}n_{a}n_{e},$$

where $n_{a}$ and $n_{e}$ are the number densities of neutral atoms and electrons, respectively. The ionization and recombination rates (in $m^{3}\;s^{-1}$) are given based on a semi-empirical model for the ionization of hydrogen by electron impact that assumes ionization from the ground state (1 s) [24,25]

$$K_{I}=2.34\times 10^{-14}X^{-1/2}\;e^{-X}$$

and

$$K_{R}=5.2\times 10^{-20}\sqrt{X}(0.4288+0.5\ln X+0.4698X^{-1/3}),$$

where $X=0.6\epsilon_{i}/T(eV)$ and $\epsilon_{i}=13.6$ eV is the ionization potential of the hydrogen atom with an electron in the ground state. These rates do not include photo-ionization or ionization from excited states, which are known to be important in the chromosphere. As the physical mechanism described in the present study requires hot electrons involved in ionization and cool electrons involved in recombination, we will differentiate between the dimensionless quantity X in $K_{I}$ and $K_{R}$ and label them as $X_{I}$ and $X_{R}$, respectively. The variation of the two rates for a large range of temperatures is shown in figure 1. While the recombination rate is fairly constant over the investigated temperature range, the ionization rate increases with temperature. For lower temperatures, recombination is indeed larger, while for a hot plasma, ionization is the dominant mechanism.

The system of equations (2.1) and (2.2) can be reduced by eliminating the neutral number density in favour of electron number density. We assume that the plasma is weakly ionized and the physical process leads to a small change in the number of neutral atoms, therefore we write $n_{a}=n_{a0}+n_{a1}$, with $n_{a0}$ being a time-averaged constant number density and $n_{a1}\ll n_{a0}$. Collecting terms according to the powers of $n_{a1}$, we obtain that the temporal evolution of electron density is described by

2.3 $$\frac{d^{2}n_{e}}{dt^{2}}+n_{a0}n_{e}^{2}(3K_{I}K_{R}+K_{I}^{2})-K_{I}^{2}n_{a0}^{2}n_{e}=O(n_{a}).$$

In addition, in the above equation, we have neglected terms that are proportional to $n_{e}/n_{a0}$. Let us write equation (2.3) in dimensionless form and denote $\alpha_{1}=3K_{I}K_{R}+K_{I}^{2}$ and $\alpha_{2}=K_{I}^{2}$. We write the number density of electrons as $n_{e}=n_{e0}+n_{e1}=n_{e0}(1+r)$, where $n_{e0}$ is a time-averaged constant density of electrons and $n_{e1}$ a perturbation of arbitrary magnitude. Furthermore, we introduce $\omega_{I}^{2}=\alpha_{1}n_{a0}n_{e0}=n_{a0}n_{e0}(3K_{I}K_{R}+K_{I}^{2})$ and $x=\alpha_{2}n_{a0}/(\alpha_{1}n_{e0})$. Using the new notation, the governing equation reduces to

2.4 $$\frac{1}{\omega_{I}^{2}}\frac{d^{2}r}{dt^{2}}+r^{2}+(2-x)r+1-x=0.$$

Equation (2.4) is a nonlinear ordinary differential equation that admits periodic nonlinear wave solutions (known as cnoidal waves) that can be expressed in terms of elliptic functions. To reduce this equation to a form that can be solved, let us multiply the whole equation by dr/dt and integrate with respect to t, leading to

2.5 $${\left(\omega_{I}^{-1}\frac{dr}{dt}\right)}^{2}+\frac{2}{3}r(r-r_{1})(r-r_{2})=0,$$

where

2.6 $$r_{1,2}=-\frac{3}{4}\left[(2-x)\pm \sqrt{\left(x+2)(x-2/3\right)}\right].$$

**Figure 2.**
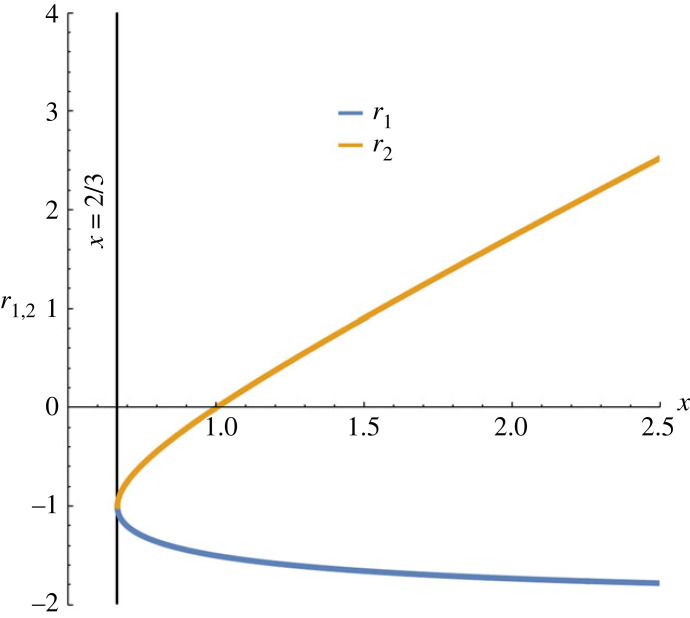
The variation of the two values $r_{1}$ and $r_{2}$ given by equation (2.6) as a function of the variable x.

It is well known that a differential equation of the type $\text{dq}\;{\text{dz}}^{-1}=\pm \sqrt{G(q)}$ (similar to equation (2.5)) admits periodic nonlinear wave solutions expressed in terms of elliptical functions if G(q) is a cubic or quartic function with distinct roots and at least two of them are real with G(q) being negative between them. That is why equation (2.5) can describe oscillatory motion in a potential well where the function r(t) is negative. The variation of the two quantities $r_{1}$ and $r_{2}$ with respect to the variable x is shown in figure 2. Depending on the value of the variable x, we can distinguish two different cases:

Case 1: 2/3<x<1. This case involves a moderate temperature difference between the ionizing and recombining electrons and a moderate ratio of the neutral and electron number densities for which the weakly ionized limit is not always satisfied. From figure 2, it is obvious that in this case $r_{1,2}<0$ and the solution of the differential equation (2.5) can be written as

2.7 $$r(t)=r_{2}{\text{sn}}^{2}\left(\sqrt{\frac{|r_{1}|}{6}}\omega_{I}t;k_{1}\right),$$

where sn(x;k) is Jacobi’s elliptic sine function and $k_{1}=\sqrt{r_{2}/r_{1}}$ is the elliptic modulus. Using the properties of elliptic functions, the frequency of the ionization-recombination wave is

2.8 $$\omega_{ir}=\frac{\omega_{I}}{2K(k_{1})}\sqrt{\frac{|r_{1}|}{6}},$$

with $K(k_{1})$ being the complete elliptic integral of the first kind. For a more intuitive understanding of the period of the ionization-recombination wave, we can consider the connection between the complete elliptic integrals and hypergeometric functions [26]. Accordingly, we have

$$K(\zeta )=\frac{\pi }{2}{\;}_{2}F_{1}\left(\frac{1}{2},\frac{1}{2};1;\zeta \right).$$

It is clear that $r_{2}/r_{1}\ll 1$, therefore using the series expansion of the hypergeometric function, we have

2.9 $$\omega_{ir}=\frac{\omega_{I}}{\pi }\frac{\sqrt{|r_{1}|/6}}{1+\sqrt{r_{2}/r_{1}}/4+O(k_{1})}.$$

**Figure 3.**
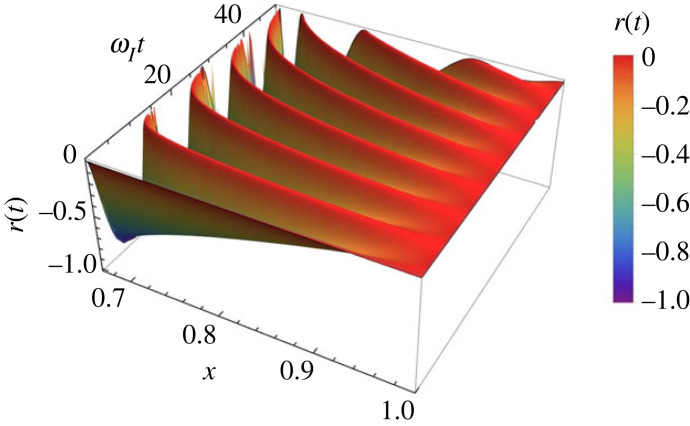
The variation of the dimensionless perturbation of the electron number density, r(t), with respect to dimensionless time, $\omega_{I}t$, and the variable x corresponding to case 1, i.e. when 2/3<x<1.

The variation of the dimensionless electron density, r(t), with respect to the variable x and time t is shown in figure 3. The amplitude of oscillations in r(t) (the dimensionless electron number density) is higher for smaller values of x, i.e. in regimes that correspond to a more ionized plasma. The amplitude of oscillations suggests that changes are nonlinear, while a more linear behaviour is obtained for values of x closer to 1.

**Figure 4.**
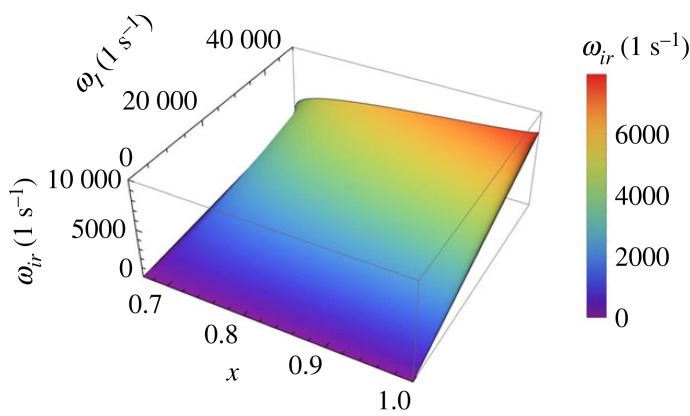
The variation of the frequency of the ionization-recombination wave, $\omega_{ir}$, with respect to the dimensionless variable x and frequency $\omega_{I}$ corresponding to case 1.

The variation of the frequency of the ionization-recombination, $\omega_{ir}$, as given by equation (2.8) is displayed in figure 4. This variation shows that the frequency of these waves (for given value of temperature) is smaller for the case when the plasma is strongly ionized and the frequency increases with decreasing the equilibrium electron number density, i.e. when the plasma tends more towards a weakly ionized state. Given the limitations that apply for this case, and the typical values of particle number densities and temperatures relevant for the solar atmosphere, the obtained solutions remain to a large extent mainly of mathematical interest.

Case 2: x>1 that corresponds to $r_{1}<0<r_{2}$. In this case, the restrictions on number densities and temperatures do not apply, and the results are more relevant to solar atmospheric conditions. Here, the solution of the differential equation reduces to

2.10 $$r(t)=\frac{r_{1}r_{2}{\text{sn}}^{2}(Z_{1};k_{2})}{r_{1}-r_{2}+r_{2}{\text{sn}}^{2}(Z_{1};k_{2})},$$

where the function $Z_{1}$ and the elliptic modulus, $k_{2}$, are defined as

$$Z_{1}=\frac{\omega_{I}t}{2}{\left[\left(x-\frac{2}{3}\right)(x+2)\right]}^{1/4}\;\mathrm{and}\;k_{2}={\left(\frac{r_{2}}{r_{2}-r_{1}}\right)}^{1/2}.$$

Again, using the properties of the elliptic function, the frequency of the ionization-recombination wave is given by

2.11 $$\omega_{ir}=\frac{\omega_{I}}{4K(k_{2})}{\left[\left(x-\frac{2}{3}\right)(x+2)\right]}^{1/4}.$$

**Figure 5.**
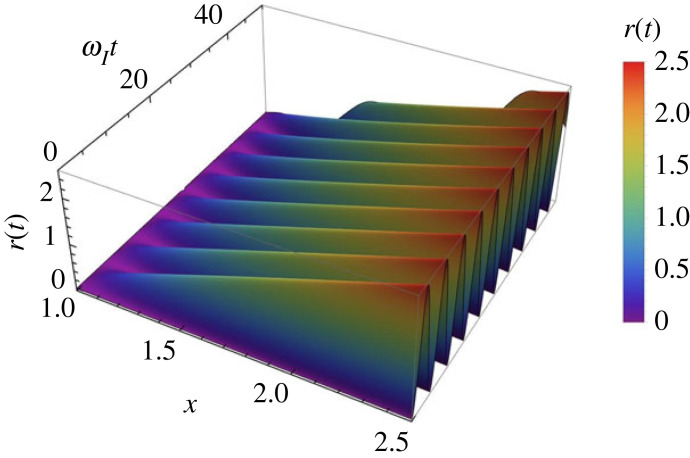
The same as in figure 3, but here we show the variation of the frequency of the ionization-recombination wave for case 2, i.e. when x>1.

**Figure 6.**
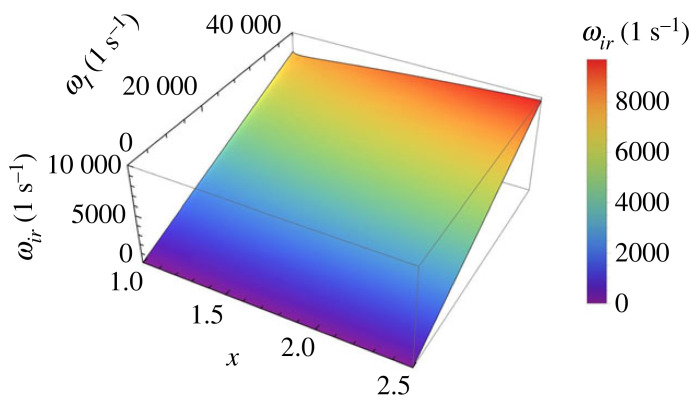
The variation of the frequency of the ionization-recombination wave, $\omega_{ir}$, with respect to the dimensionless variable x and the frequency $\omega_{I}$ corresponding to case 2.

**Figure 7.**
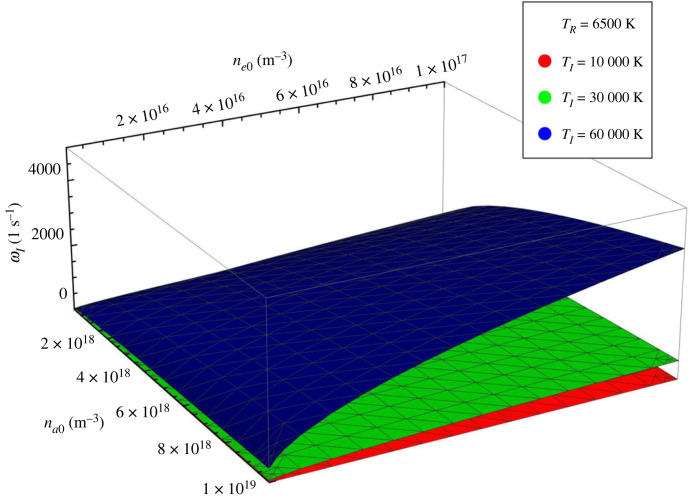
The variation of $\omega_{I}$ with respect to electron and neutral number densities for a particular recombination temperature ($T_{R}=6500$ K) and three different ionization temperatures, shown by different colours: $T_{I}=10^{4}$ K (red), $T_{I}=3\times 10^{4}$ K (green) and $T_{I}=6\times 10^{4}$ K (blue).

As before, we can use the connection between the complete elliptical integral and the ${\;}_{2}F_{1}$ hypergeometric function. It can be shown that the elliptical modulus, $k_{2}$, always remains smaller than 1, so the series expansion of the hypergeometric function leads to an algebraic expression of the ionization-recombination frequency of the form

$$\omega_{ir}=\frac{\omega_{I}}{2\pi }\frac{{\left[(x+2)(x-2/3)\right]}^{1/4}}{1+k_{2}/4+O(k_{2}^{2})}.$$

The variation of the quantity r(t) with respect to the variable x and the dimensionless parameter $\omega_{I}t$ is shown in figure 5 and it is clear that the amplitude of waves increases with the value of x. Similar to the previous case, the variation of the frequency of the ionization-recombination wave in terms of the dimensionless quantity x and the frequency $\omega_{I}$ is shown in figure 6 and this quantity shows a small increase with the value of x and shows smaller amplitudes (i.e. closer to a linear description) for values of x closer to 1. It is interesting to note that at x≈1 the frequency of the ionization-recombination waves in the two cases become equal and this value is given by

$$\omega_{ir}=\frac{\omega_{I}}{4K(0)}=\frac{\omega_{I}}{2\pi }.$$

A special case constitutes the limit when |x−1|≪1, which would also imply that r≪1, so we are dealing with a linear approach. In this case, the governing equation (2.4) reduces to a simple small amplitude harmonic non-homogeneous equation with frequency $\omega_{I}$ and the time-dependent electron density becomes

2.12 $$n_{e}(t)=n_{e0}+n_{e0}\epsilon \cos\omega_{I}t,$$

where we used the notation ϵ=1−r and the frequency $\omega_{I}$ was defined earlier and depends on the ionization/recombination rate and the equilibrium number densities of ions and neutrals. In this limit, $\omega_{I}$ represents the frequency of the ionization-recombination waves. The temporal change in the number density of electrons owing to ionization-recombination processes will influence the other waves that can appear in the plasma and this influence can appear as a parametric resonance between the two oscillations. The variation of $\omega_{I}$ with respect to the electron and neutral number densities is presented in figure 7 for a fixed recombination temperature of 6500 K and for three different values of the ionization temperature. These results should be interpreted carefully, as one of the key requirements of the model discussed in our study is the weakly ionized character of the plasma, so we would always need to choose a combination of number densities such that $n_{a0}>n_{e0}$. Clearly, the largest values of the ionization-recombination frequency is obtained for very large ionization temperature. Finally, we should mention that for values of x<2/3 the solutions of the governing equation (2.5) are aperiodical.

## 3. Parametric resonance of Alfvén waves

Let us consider that the partially ionized plasma is permeated by a homogeneous magnetic field oriented in the z-direction. In this environment charged particles and neutrals collide, providing an effective channel for momentum and energy exchange between the two species. Alfvén waves will propagate along the z-axis and will be polarized along the y-axis. Since we assumed that the plasma is quasi-neutral, $n_{i0}=n_{e0}$, the equilibrium ion number density will be consistently replaced by the equilibrium electron number density. In order to simplify the mathematical description, we consider a reference frame where neutrals are at rest. As a result, the governing equations of the charged species are given by

3.1 $$m_{i}n_{e}(t)\frac{\partial v_{iy}}{\partial t}=\frac{B_{0}}{\mu_{0}}\frac{\partial b_{y}}{\partial z}-m_{i}n_{e}(t)(\nu_{i}+n_{e}(t)K_{R})v_{iy}$$

and

3.2 $$\frac{\partial b_{y}}{\partial t}=B_{0}\frac{\partial v_{iy}}{\partial t},$$

where $v_{iy}$ and $b_{y}$ are the velocity and magnetic field perturbations associated with the charged species, the electron (and consequently the ion) number density, $n_{e}$, is given by equation (2.12) and $\mu_{0}$ is the permeability of free space. Having considered the neutral species as immobile, the quantity $\nu_{i}$ in equation (3.1) refers to the inverse of the mean free time of ions, i.e. the average of time between two collisions. For simplicity, we consider that the collisional frequency, $\nu_{i}$ is a constant quantity. Considering immovable neutrals, we eliminate the entropy mode, a non-propagating, purely damped, solution of the dispersion relation that exists only when collisions are taken into account. The term on the right-hand side of equation (3.1) that contains the recombination rate $K_{R}$ denotes the loss of momentum of ions caused by recombination.

Assuming that perturbations are proportional to $e^{ikz}$, the above system can be reduced to a single ordinary differential equation for the velocity perturbation:

3.3 $$\frac{d^{2}v_{iy}}{dt^{2}}+a(t)\frac{dv_{iy}}{dt}+b(t)v_{iy}=0,$$

where the time-dependent coefficients a(t) and b(t) are given by

$$a(t)=\nu_{i}+n_{e0}K_{R}-\epsilon (\omega_{I}\sin\omega_{I}t-\delta \cos\omega_{I}t$$

and

$$b(t)=k^{2}v_{A}^{2}-\epsilon [k^{2}v_{A}^{2}\cos\omega_{i}t+\omega_{I}(\nu_{i}+2\delta )\sin\omega_{I}t],$$

where $v_{A}=B_{0}/\sqrt{m_{i}n_{e0}\mu_{0}}$ is the Alfvén speed, $\delta =n_{e0}K_{R}$ and all terms $O(\epsilon^{2})$ have been neglected. This equation is a Mathieu-type differential equation that can also be written as a the well-known Hill differential equation by introducing a new function so that

3.4 $$V_{iy}=v_{iy}\exp\left(\frac{1}{2}\int a(t)\;dt\right).$$

As a result, the governing equation for Alfvén waves reduces to a Hill-type differential equation of the form

3.5 $$\frac{d^{2}V_{iy}}{dt^{2}}+\Omega^{2}(t)V_{iy}=0,$$

where now

3.6 $$\begin{array}{rl} \Omega^{2}(t) & \;=b(t)-\frac{1}{2}\frac{da(t)}{dt}-\frac{1}{4}a^{2}(t)=k^{2}v_{A}^{2}-\frac{1}{4}{\left(\nu_{i}+\delta \right)}^{2}\\ & \;\;-\epsilon \cos(\omega_{I}t)\left[k^{2}v_{A}^{2}-\frac{\omega_{I}^{2}}{2}+\frac{\delta }{2}(\nu_{i}+\delta )\right]-\epsilon \omega_{I}\sin(\omega_{I}t)\left(\frac{\nu_{i}}{2}+\delta \right)+O(\epsilon^{2}). \end{array}$$

The coefficient of this equation is periodic, i.e. Ω(t+T)=Ω(t) and the above equation is invariant to the transformation t→t+T. This equation is known to be susceptible to parametric resonance and this occurs when the pumping frequency (the frequency of the ionization-recombination waves), $\omega_{I}$, satisfies the condition $\omega_{I}=2kv_{A}/m+\beta$, with β≪1 being the resonance detuning factor. The most intensive form of parametric resonance occurs when m=1, therefore we investigate the case when $\omega_{I}=2kv_{A}+\beta$.

Let us return to equation (3.3), which is a modified Mathieu differential equation. When ϵ=0 the equation (now with constant coefficients) describes the temporal variation of the eigenfunction connected to Alfvén waves and, provided $kv_{A}>{\tilde{\nu }}_{i}/2$, the solution can be simply written as

$$v_{iy}=e^{-{\tilde{\nu }}_{i}t/2}\left(A\cos\sqrt{k^{2}v_{A}^{2}-{\tilde{\nu }}_{i}^{2}/4}t+B\sin\sqrt{k^{2}v_{A}^{2}-{\tilde{\nu }}_{i}^{2}/4}t\right),$$

where ${\tilde{\nu }}_{i}=\nu_{i}+\delta$. This result confirms the standard knowledge on linear oscillators with damping, i.e. the ion-neutral damping and the loss of momentum caused by recombination cause a decaying amplitude in time and a reduction of the frequency of Alfvén waves provided the natural frequency of Alfvén waves, $kv_{A}$, is greater than $(\nu_{i}+\delta )/2$. If the above condition is not satisfied, the solution of equation (3.3) is represented by a non-propagating, exponentially decaying signal in time. The condition imposed for this solution also means that Alfvén waves will be able to propagate as long as $k>2{\tilde{\nu }}_{i}/v_{A}$, so collisions act as a filter, only wavelengths smaller than $4\pi v_{A}/{\tilde{\nu }}_{i}$ will propagate. From now on, for simplicity, we denote the natural frequency of Alfvén waves by $\omega_{n}$.

In what follows, for an arbitrary value of ϵ≪1 we use the multiple scale analysis method [27,28] to study the resonant coupling between ionization-recombination waves and Alfvén waves. According to this method, the temporal evolution of the eigenfunction contains two distinct behaviours: one is connected to the periodicity of the wave, while the other one is connected to the damping in time of the wave. That is why we introduce a fast and a slow time variable such that $\xi =\omega_{I}t$ and $\eta =\epsilon \omega_{I}t$. Since the equation is linear, no stretching variable is needed. Although transforming an ordinary differential equation into a partial differential equation may look counterintuitive, this method allows us to simplify the mathematics by considering the dominant physical effect. As a result, the governing equation (3.3) transforms into

3.7 $$\begin{array}{rl} & \frac{\partial^{2}v_{iy}}{\partial \xi^{2}}+2\epsilon \frac{\partial^{2}v_{iy}}{\partial \xi \partial \eta }+\epsilon^{2}\frac{\partial^{2}v_{iy}}{\partial \eta^{2}}+\left(\frac{{\tilde{\nu }}_{i}}{\omega_{I}}-\epsilon \sin\xi +\frac{\delta }{\omega_{I}}\cos\xi \right)\left(\frac{\partial v_{iy}}{\partial \xi }+\epsilon \frac{\partial v_{iy}}{\partial \eta }\right)\\ & \;\;\;+\frac{\omega_{n}^{2}}{\omega_{I}^{2}}v_{iy}-\epsilon \frac{\omega_{n}^{2}}{\omega_{I}^{2}}\cos\xi v_{iy}-\epsilon \left(\frac{{\tilde{\nu }}_{i}}{\omega_{I}}+\frac{2\delta }{\omega_{I}}\right)\sin\xi v_{iy}=0. \end{array}$$

In what follows, we denote by α the ratio of the natural frequency and the pumping frequency, i.e. $\alpha =\omega_{n}/\omega_{I}$. Next, we expand the eigenfunction $v_{iy}$ into power series, such that

$$v_{iy}(\xi ,\eta )=v_{iy}^{\left(0\right)}(\xi ,\eta )+\epsilon v_{iy}^{\left(1\right)}(\xi ,\eta )+\cdots .$$

In this approach, the dissipation is a second-order effect, therefore we rescale the collisional and recombination frequencies such that ${\hat{\nu }}_{i}=\epsilon {\tilde{\nu }}_{i}$ and $\hat{\delta }=\epsilon \delta$. In the first-order approximation ($O(\epsilon^{0}$) terms), equation (3.7) reduces to

3.8 $$\frac{\partial^{2}v_{iy}^{\left(0\right)}}{\partial \xi^{2}}+\alpha^{2}v_{iy}^{\left(0\right)}=0.$$

The solution of this second-order differential equation is of the form

3.9 $$v_{iy}^{\left(0\right)}=A_{0}(\eta )\cos\alpha \xi +B_{0}(\eta )\sin\alpha \xi ,$$

where the η dependence of the coefficient functions $A_{0}$ and $B_{0}$ means that these coefficients are slowly varying functions of t.

Collecting terms O(ϵ), the second-order approximation of equation (3.7) leads to an equation of the form

3.10 $$\frac{\partial^{2}v_{iy}^{\left(1\right)}}{\partial \xi^{2}}+\alpha^{2}v_{iy}^{\left(1\right)}=-2\frac{\partial^{2}v_{iy}^{\left(0\right)}}{\partial \xi \partial \tau }-\left(\frac{{\hat{\nu }}_{i}}{\omega_{I}}-\sin\xi +\frac{\hat{\delta }}{\omega_{I}}\cos\xi \right)\frac{\partial v_{iy}^{\left(0\right)}}{\partial \xi }+\alpha^{2}\cos\xi v_{iy}^{\left(0\right)}.$$

The left-hand side of the above equation is similar to the equation obtained in the first-order approximation, while the right-hand side terms are all expressed in terms of the unknown function $v_{iy}^{\left(0\right)}$. Using the particular expression of $v_{iy}^{\left(0\right)}$ (see equation (3.9)) and some fundamental trigonometric identities, the second-order approximation can be written as

3.11 $$\begin{array}{rl} \frac{\partial^{2}v_{iy}^{\left(1\right)}}{\partial \xi^{2}}+\alpha^{2}v_{iy}^{\left(1\right)} & \;=\alpha \left[2A_{0}^{\prime }(\eta )+\frac{{\hat{\nu }}_{i}}{\omega_{I}}A_{0}(\eta )\right]\sin\alpha \xi -\alpha \left[2B_{0}^{\prime }(\eta )+\frac{{\hat{\nu }}_{i}}{\omega_{I}}B_{0}(\eta )\right]\cos\alpha \xi \\ & \;\;-\frac{\alpha A_{0}(\eta )}{2}[\cos\xi (\alpha -1)+\cos\xi (\alpha +1)]+\frac{\alpha B_{0}(\eta )}{2}[\sin\xi (\alpha +1)-\sin\xi (\alpha +1)]\\ & \;\;-\frac{\alpha }{2}\left(\frac{\hat{\delta }}{\omega_{I}}B_{0}(\eta )-\alpha A_{0}(\eta )\right)[\cos\xi (\alpha +1)-\cos\xi (\alpha -1)]\\ & \;\;+\frac{\alpha }{2}\left(\frac{\hat{\delta }}{\omega_{I}}A_{0}(\eta )+\alpha B_{0}(\eta )\right)[\sin\xi (\alpha -1)+\sin\xi (\alpha +1)]. \end{array}$$

Secular growth in the solution of equation (3.11) is given by the first two terms of the right-hand side of the above equation. Indeed, choosing

$$A_{0}(\eta )=A_{0}\exp\left(-\frac{{\hat{\nu }}_{i}\alpha }{2\omega_{I}}\eta \right)\;\mathrm{and}\;B_{0}(\eta )=B_{0}\exp\left(-\frac{{\hat{\nu }}_{i}\alpha }{2\omega_{I}}\eta \right),$$

with $A_{0}$ and $B_{0}$ two arbitrary constant amplitudes, these terms vanish; however this solution would not take into account the presence of the driving trigonometric term in the original Mathieu equation. Instead, if we choose α=1/2, additional resonant terms can be recovered and, in this case, the right-hand side of the equation can be written as

$$\begin{array}{rl} & \frac{1}{2}\left[2A_{0}^{\prime }(\eta )+\frac{{\hat{\nu }}_{i}}{\omega_{I}}A_{0}(\eta )\right]\sin\frac{\xi }{2}-\frac{1}{2}\left[2B_{0}^{\prime }(\eta )+\frac{{\hat{\nu }}_{i}}{2\omega_{I}}B_{0}(\eta )\right]\cos\frac{\xi }{2}\\ & \;\;-\frac{A_{0}(\eta )}{4}\left[\cos\frac{\xi }{2}-\cos\frac{3\xi }{2}\right]+\frac{B_{0}(\eta )}{4}\left[\sin\frac{3\xi }{2}+\sin\frac{\xi }{2}\right]\\ & \;\;+\frac{1}{4}\left(\frac{\hat{\delta }}{\omega_{I}}A_{0}(\eta )+\frac{B_{0}(\eta )}{2}\right)\left[\sin\frac{3\xi }{2}-\sin\frac{\xi }{2}\right]\\ & \;\;-\frac{1}{4}\left(\frac{\hat{\delta }}{\omega_{I}}B_{0}(\eta )-\frac{A_{0}(\eta )}{2}\right)\left[\cos\frac{3\xi }{2}+\cos\frac{\xi }{2}\right]. \end{array}$$

The choice of α=1/2 covers the case of exact resonance, i.e. the case when the pumping frequency $\omega_{I}$ is exactly twice the natural frequency of Alfvén waves, $kv_{A}$. However, the parametric resonance occurs for a larger frequency interval, meaning that we write the quantity α in the form of an expansion given by

$$\alpha =\frac{1}{2}+\epsilon \alpha_{1}+\epsilon^{2}\alpha_{2}+\cdots ,$$

where terms O(ϵ) denote the detuning of the system from exact resonance. In this case, equation (3.11) becomes

3.12 $$\begin{array}{rl} \frac{\partial^{2}v_{iy}^{\left(1\right)}}{\partial \xi^{2}}+\frac{1}{4}v_{iy}^{\left(1\right)} & \;=-2\frac{\partial^{2}v_{iy}^{\left(0\right)}}{\partial \xi \partial \tau }-\left(\frac{{\hat{\nu }}_{i}}{\omega_{I}}-\sin\xi +\frac{\hat{\delta }}{\omega_{I}}\cos\xi \right)\frac{\partial v_{iy}^{\left(0\right)}}{\partial \xi }+\frac{1}{4}\cos\xi v_{iy}^{\left(0\right)}-\alpha_{1}v_{iy}^{\left(0\right)}. \end{array}$$

Again, using the particular form of $v_{iy}^{\left(0\right)}$, imposing the same conditions as before regarding the vanishing of resonant terms with the neglect of non-resonant terms (e.g. terms containing cos⁡3ξ/2 and sin⁡3ξ/2), we arrive at the system of coupled equations

3.13 $$\left\{ \begin{array}{l} \frac{dA_{0}(\eta )}{d\eta }=-\frac{{\hat{\nu }}_{i}}{2\omega_{I}}A_{0}(\eta )+\frac{\hat{\delta }}{4\omega_{I}}A_{0}(\eta )+\left(\alpha_{1}-\frac{1}{8}\right)B_{0}(\eta ),\\ \frac{dB_{0}(\eta )}{d\eta }=-\frac{{\hat{\nu }}_{i}}{2\omega_{I}}B_{0}(\eta )-\frac{\hat{\delta }}{4\omega_{I}}B_{0}(\eta )-\left(\alpha_{1}+\frac{1}{8}\right)A_{0}(\eta ). \end{array}\right.$$

The above homogeneous linear system with constant coefficients can be solved by assuming a solution of the form $A_{0}(\eta )=A_{0}\;e^{\lambda \eta }$ and $B_{0}(\eta )=B_{0}\;e^{\lambda \eta }$. The compatibility condition of the system of equations requires that the determinant constructed from the coefficients of the unknown amplitudes $A_{0}$ and $B_{0}$ vanishes, i.e.

3.14 $$\left|\begin{array}{cc} \frac{{\hat{\nu }}_{i}}{2\omega_{I}}-\frac{\hat{\delta }}{4\omega_{I}}+\lambda & -\alpha_{1}+\frac{1}{8}\\ \alpha_{1}+\frac{1}{8} & \frac{{\hat{\nu }}_{i}}{2\omega_{I}}+\frac{\hat{\delta }}{4\omega_{I}}+\lambda \end{array}\right|=0,$$

which means that

3.15 $$\lambda =-\frac{{\hat{\nu }}_{i}}{2\omega_{I}}\pm \sqrt{\frac{1}{64}-\alpha_{1}^{2}+\frac{{\hat{\delta }}^{2}}{16\omega_{I}^{2}}}.$$

The transition between stable and unstable behaviour is determined by the condition λ=0, so the transition curves satisfy the condition

3.16 $$\alpha_{1}=\pm \frac{1}{2}\sqrt{\frac{1}{16}-{\left(\frac{{\hat{\nu }}_{i}}{\omega_{I}}\right)}^{2}+{\left(\frac{\hat{\delta }}{2\omega_{I}}\right)}^{2}}.$$

With this value of $\alpha_{1}$, the expression of α becomes

3.17 $$\begin{array}{rl} \alpha & \;=\frac{1}{2}\pm \frac{\epsilon }{2}\sqrt{\frac{1}{16}-{\left(\frac{{\hat{\nu }}_{i}}{\omega_{I}}\right)}^{2}+{\left(\frac{\hat{\delta }}{2\omega_{I}}\right)}^{2}}+O(\epsilon^{2})=\frac{1}{2}\pm \frac{1}{2}\sqrt{\frac{\epsilon^{2}}{16}-{\left(\frac{{\tilde{\nu }}_{i}}{\omega_{I}}\right)}^{2}+{\left(\frac{\delta }{2\omega_{I}}\right)}^{2}}+O(\epsilon^{2}). \end{array}$$

The above relation stipulates that the stable/unstable behaviour of the system is regulated by the ratio of the collisional and pumping frequency, as well as by the ratio of the recombination rate and pumping frequency. At resonance, the natural frequency of Alfvén waves (in dimensional form) can be written as

3.18 $$\omega_{n}=\frac{\omega_{I}}{2}\pm \frac{\omega_{I}}{2}\sqrt{\frac{\epsilon^{2}}{16}-{\left(\frac{{\tilde{\nu }}_{i}}{\omega_{I}}\right)}^{2}+{\left(\frac{\delta }{2\omega_{I}}\right)}^{2}}+O(\epsilon^{2}),$$

and it is clear that Alfvén waves remain stable as long as

$${\tilde{\nu }}_{i}<\frac{1}{2}\sqrt{\frac{\epsilon^{2}\omega_{I}^{2}}{4}+\delta^{2}}.$$

Let us finally investigate the parametric domain where the system is stable or unstable. For that we use equation (3.17) and introduce the notation

$$h=\sqrt{{\left(\frac{{\tilde{\nu }}_{i}}{\omega_{I}}\right)}^{2}-{\left(\frac{\delta }{2\omega_{I}}\right)}^{2}}.$$

**Figure 8.**
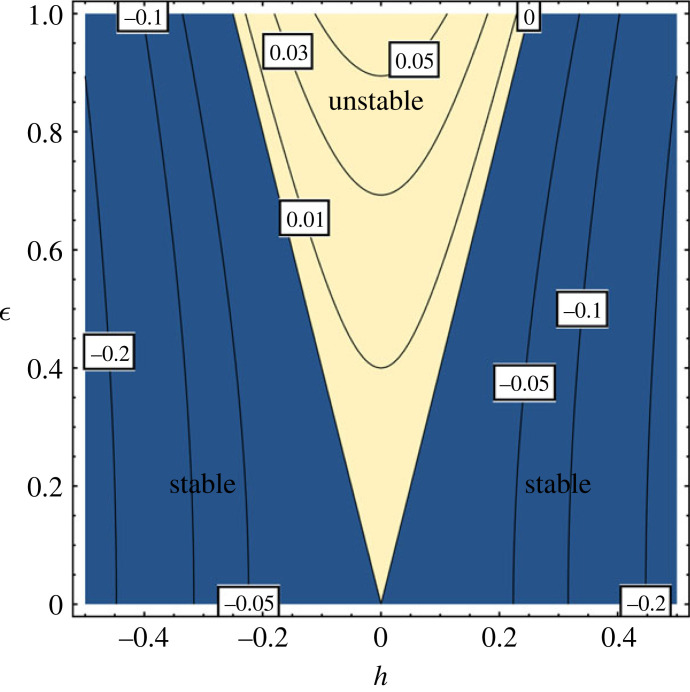
The Floquet tongue shows the stability and instability regions of the first-order approximation of Alfvén waves parametrically coupled with the ionization-recombination waves.

This parameter contains information about the physical mechanisms that affect the propagation of Alfvén waves, i.e. the collisions of ions with neutrals and the loss of ion momentum owing to recombination effects. The stability of the system for a full range of parameters can be investigated by constructing the first Floquet tongue of the system (figure 8). Equation (3.18) predicts that for a given value of h, there is a minimum value of ϵ which is required for instability to occur. In figure 8, the central region corresponds to an unstable behaviour, while outside this region the system is stable. With the increase in the value of h the threshold of ϵ where the system is unstable is modified (the tongue shifts up, the regions bounded by the parabolas), and the action of the collisions and recombination of ions has a stabilizing effect on the parametric instability, in line with the conclusions regarding the role collisions between particles obtained for other types of instabilities [6,29,30]. The lines at ϵ=±4h denote the instability thresholds for various values of the parameter h, and it is clear that the region of instability shrinks with the increase in the value of this parameter. The way the quantity h is defined suggests that collisional and recombination effects can contribute to the development of instability.

### (a) Application to the solar atmosphere

The partially ionized solar atmosphere serves as a perfect environment where the ionization-recombination wave mechanism described above and the associated parametric coupling of these waves to other MHD modes can occur. The theoretical model presented in our study involves some requirements, however, these are realistic. For instance, the process of periodic ionization and recombination processes require first that in a given plasma volume the temperature of electrons is very high. There are numerous examples in the solar atmosphere of energetic (hot) electrons in the forms of jets or spicules that could originate from, e.g. reconnection events [31–33].

The analysis of the possible solutions of the governing equation (2.5) translated to solar atmospheric conditions (number densities, temperature) reveal that the solutions of the first case (2/3<x<1) are, to a very large extent, mainly of mathematical interest, while realistic conditions are satisfied for the situation presented as case 2, i.e. when the dimensionless variable x is larger than 1. Here, depending on the number density of charged particles and temperature, the frequency of the ionization-recombination waves can reach values that are comparable to the collisional frequency between the massive particles of the problem relevant in the solar chromosphere.

A separate discussion was presented for the case when the plasma parameters are very close to the transition point x=1 between the two regimes; here the ionization-recombination waves become linear and an easier estimation of their characteristics is possible. Using typical values of the VAL III C model [22], and considering the temperature of the ionizing electrons to be $T_{i}=6\times 10^{4}\;\text{K}$, the temperature of recombining electrons $T_{r}=6500$ K with $n_{a0}=10^{18}\;m^{-3}$, $n_{e0}=6.5\times 10^{16}\;m^{-3}$, we obtain $\omega_{I}\approx 10^{3}$ Hz, i.e. a period of the ionization-recombination wave of $\approx \;6.2\times 10^{-3}\;\text{s}$. This value of the frequency is smaller than the collisional frequency between ions and neutrals corresponding to these plasma parameters ($\approx 9\times 10^{4}$ Hz). This value of frequency currently cannot be measured with the current observational facilities (possibly visible in radio wavelength, [34,35]); however to an observer, the presence of these waves would appear as periodically glowing plasma regions, or striations. Striation formation owing to energetic electron jets are frequently observed in plasma discharge tubes [36–38].

## 4. Conclusion

This study investigated the properties of ionization-recombination waves that can appear in the weakly ionized solar atmospheric plasma in the presence of a hot population of electrons and the parametric coupling of these waves to the Alfvén waves that can propagate in such plasmas.

Collisional ionization and recombination processes give rise to a temporal variation of electron (and subsequently proton) number density that manifests itself in the form of a non-propagating wave present in this plasma. Our results show that depending on the relative magnitude of the ionization and recombination rates (through the value of the dimensionless parameter x) the frequency of the ionization-recombination waves behave differently and their frequency is given in terms of the complete elliptical integrals of the first kind.

The ionization-recombination waves, through the associated time-dependent number density, can parametrically couple with the Alfvén waves that propagate along a unidirectional magnetic field. For simplicity, we assumed a coordinate system attached to neutrals, which allowed us to simplify the governing equation for Alfvén waves. The governing equations have been reduced to a Mathieu-type equation (or the associated Hill’s equation) and the solution has been sought by means of a multiple scale technique. As expected, the maximum efficiency of coupling occurs when the frequency of the ionization-recombination waves is approximately twice the natural frequency of Alfvén waves. Our results suggest that the unstable amplitude growth of Alfvén waves is influenced by collisions and loss of momentum caused by recombination.

The work has several limitations. First, we restricted ourselves to ionization and recombination processes that involve collisions. In the optically thick partially ionized solar plasma, photoionization can play an equally important role; however, in here, this process was neglected. In addition, we limited our analysis to the coupling involving Alfvén waves, given their simple governing equation. As the ionization-recombination waves imply a time-dependent equilibrium density, other waves will also be affected. We intend to expand in the future the present research to the investigation of the development of parametric instability for slow and fast magnetoacoustic waves.

## Data Availability

This article has no additional data.
